# Are platinum agents, paclitaxel and irinotecan effective for clear cell carcinoma of the ovary? DNA damage detected with γH2AX induced by anticancer agents

**DOI:** 10.1186/1757-2215-5-16

**Published:** 2012-06-12

**Authors:** Eriko Takatori, Tadahiro Shoji, Seisuke Kumagai, Takashi Sawai, Akira Kurose, Toru Sugiyama

**Affiliations:** 1 Department of Obstetrics and Gynecology, Iwate Medical University School of Medicine, 19-1, Uchimaru, Morioka, Iwate, 020-8505, Japan; 2 Department of Pathology, Iwate Medical University School of Medicine, Uchimaru, Morioka, Iwate, 020-8505, Japan; 3 Department of Anatomic Pathology, Hirosaki University Graduate School of Medicine, 5, Zaifu-cho, Hirosaki, Aomori, 036-8562, Japan

**Keywords:** γH2AX, Clear cell carcinoma, Ovarian cancer, DNA damage, Apoptosis, Chemotherapy

## Abstract

**Objectives:**

Differences in the incidences and types of DNA damage induced by antitumor agents for clear cell carcinoma (CCC) were determined in 2 ovarian CCC cell lines using γH2AX.

**Material and methods:**

The antitumor activity of anticancer agents, CDDP, CBDCA, PTX and SN-38, was examined using ovarian clear cell carcinoma cultured cell lines (OVISE and RMG-I). After culture, each cell line was treated with each anticancer agent, the cells were collected, fixed, and then reacted with the anti-γH2AX antibody. γH2AX and nuclear DNA were then simultaneously detected by flow cytometry using FITC and propidium iodide, respectively, to determine γH2AX in each cell cycle phase.

**Results:**

After administration of CDDP, DNA damage was frequent in S-phase cells, while cell-cycle arrest occurred in the G1 and G2/M phases and γH2AX did not increase in CDDP-resistant cells. Sensitivities to CDDP and CBDCA differed between the two cell lines. The antitumor effect of PTX is induced by G2/M arrest, and combination treatment with CBDCA, inducing DNA damage in G2/M-phase cells, might be effective.

**Conclusions:**

This is the first study in Japan to evaluate the antitumor activity of anticancer agents by focusing on the relationship between the cell cycle and DNA damage using γH2AX as an indicator. The immunocytochemical method used in this study detects γH2AX, which indicates DNA damage even at very low concentrations and with high sensitivity. Therefore, a promising method of easily and rapidly identifying agents potentially effective against CCC.

## Introduction

Clear cell adenocarcinoma (CCC), a subtype of epithelial ovarian cancer, is less sensitive to chemotherapy and is thus classified as a refractory ovarian cancer [1]. It has been shown that a combination of carboplatin (CBDCA) and paclitaxel (PTX ), a standard therapy for ovarian cancer [2,3], is effective against serous adenocarcinoma and endometrioid adenocarcinoma, with a response rate of approximately 75%, while CCC has lower response rates ranging from 18% to 50% [4]. The incidence of CCC has been increasing and is now 25% in Japan, while that in Europe is 5-6%. As yet, no treatment for this histological subtype of ovarian cancer has been established. Histopathology remains the gold standard for classifying epithelial ovarian cancer subgroups; however, there is emerging evidence indicating different genetic and molecular profiles. Consequently, there is no international consensus regarding the necessity of establishing treatment strategies based on histological subtypes. In fact, global clinical trials of CCC and mucinous adenocarcinoma have already begun. Although which cytotoxic agents have true efficacy against CCC remains unknown, small trials in Japan and basic studies have suggested the efficacy of irinotecan (CPT-11) [5-7]. The Japanese Gynecologic Oncology Group (JGOG) started an international randomized controlled trial (RCT) of cisplatin (CDDP)/CPT-11 therapy with a control arm of CBDCA/TXL (TC) therapy (JGOG3017/GCIG); patient accrual is ongoing and approximately 560 patients had been enrolled in the trial as of July 2010. In addition, an ongoing translational study, as part of the JGOG3017/GCIG trial, also aims to establish an updated treatment strategy.

Nucleosomes, units of chromatin, consist of core histones wrapped in 146 bp of DNA and linker DNA. Core histones are octamers designated H2A, H2B, H3 and H4. Histone H2AX is a variant of histone H2A and accounts for 10-15% of all variants. When DNA damage occurs, serine 139 of histone H2AX in chromatins on both sides of a damaged site is phosphorylated by two enzymes: ataxia telangiectasia mutated (ATM) protein kinase and by ATM and Rad3 related (ATR) protein kinase [8,9]. Phosphorylated histone H2AX is called γH2AX. Dot γH2AX, which is detectable using γH2AX-specific antibody, is considered to correspond to specific DNA damage. Therefore, DNA damage can be immunocytochemically detected [10]. DNA damage in individual cells has been detected employing a single-cell DNA gel electrophoresis technique (comet assay), in which the extent and length of the comet’s tail indicate the severity of DNA damage. Recently, however, it has become apparent that phosphorylation of histone H2AX, one of the variants of the nucleosome core histone H2A, can provide a sensitive and reliable marker of DNA damage. More specifically, DNA damage, particularly that involving the formation of DNA double-strand breaks (DSBs), induces phosphorylation of histone H2AX on Ser-139; phosphorylated H2AX is defined as γH2AX. The phosphorylation takes place on H2AX molecules on both sides of DSBs along a megabase length of DNA. Although DSBs generated during DNA fragmentation in the course of apoptosis also induce γH2AX, the degree of γH2AX induction in apoptotic cells is much greater than that in primary DSBs induced by antitumor drugs or radiation. The presence of γH2AX in cells can be detected immunocytochemically in the form of distinct nuclear γH2AX immunofluorescent foci and each focus is considered to correspond to a single DSB. This immunocytochemical approach has made it possible to assay DNA damage and *in situ* repair of the chromatin of individual cells. The immunocytochemical approach is significantly more sensitive than the comet assay. The use of multi-parameter flow cytometry in measurements of γH2AX immunofluorescence allows DNA damage to be correlated with cellular DNA content and, therefore, the cell-cycle phase. Determination of the cell-cycle phase targeted by the drug is of importance in elucidating the mechanism of antitumor drug activity.

In the present study, we conducted flow cytometric bivariate analyses of γH2AX and DNA contents in two different cell lines of CCC treated with CDDP, CBDCA, PTX or CPT-11 (SN-38), which have been used in the aforementioned international clinical randomized trial targeting CCC, and examined effects of these drugs with regard to the induction of DNA damage, apoptosis and cell-cycle progression vis-à-vis the cell-cycle phase.

## Materials and methods

### Cell culture

We used two CCC cell lines (OVISE and RMG-I) were obtained from the Health Science Research Resources Bank (Osaka, Japan). OVISE was established from a patient with metastatic disease after completion of six cycles of platinum combination therapy, and was grown in dishes (Becton Drive, Franklin Lakes, NJ, USA) in RPMI1640 medium (Sigma Chemical Co., St Louis, MO, USA) with 10% fetal bovine serum. RMG-I was established from a chemotherapy-naïve patient with ascites, and was reported to be primary platinum resistant (Table 1) [11]. RMG-I was grown in dishes (Becton Drive) in Ham F-12 medium supplemented with 10% fetal bovine serum. The media for the two cell lines were supplemented with 100 U/ml penicillin and 100 μg/ml streptomycin (Meiji Seika Co., Ltd., Tokyo, Japan). All cell lines were maintained at 37°C in a humidified atmosphere of 5% CO_2_ in air.

**Table 1:** Clinical biological characteristics of the cell line

**Cell Line**	**Source**	**Histopathology**	**Pretreatment**	**Median doubling time**
OVISE	Solid metastatic	CCC	CAP × 6 courses	60 hours
RMG- 1	Ascites	CCC	No	60 hours

CCC; clear cell carcinoma

CAP; cyclophosphamide, doxorubicin, cisplatin

### Drugs

CDDP, PTX and SN-38 (CPT-11) were dissolved in dimethyl sulfoxide (DMSO, Sigma); the final concentration of DMSO in the culture medium never exceeded 0.1% (w/v). CBDCA was dissolved in phosphate-buffered saline (PBS). The concentration of each agent was set to correspond to the blood concentration at a standard clinical dose (Table 2).

**Table 2:** Minimum effective concentration (MEC)

	**Cmax**	**MEC**
PTX	10 μg/ml	50 ng/ml
CDDP	7 μg/ml	1 μg/ml
CBDCA	55 μg/ml	10 μg/ml
SN-38	30 μg/ml	1 ng/ml

Cmax; clinical maximum drug concentration.

### Immunohistochemistry

Both cells floating in the medium and the cells that remained attached after trypsinization were collected and fixed with 1% methanol-free formaldehyde (Polysciences Inc., Warrington, PA, USA) in PBS at 0 °C for 15 minutes and post-fixed with 80% ethanol for at least 2 hours at −20 °C. The fixed cells were washed twice in PBS and suspended in a 1% (w/v) solution of bovine serum albumin (BSA) (Sigma) in PBS to suppress non-specific antibody binding. The cells were then incubated in 100 μl of 1% BSA containing 1:100 diluted anti-phosphohistone H2AX (Ser-139) monoclonal antibody (Upstate, Lake Placid, NY, USA) for 2 hours at room temperature, washed twice with PBS and resuspended in 100 μl of 1:20 diluted fluorescein isothiocyanate (FITC)-conjugated F(ab’)^2^ fragment of goat anti-mouse immunoglobulin (Dako, Glostrup, Denmark) for 30 minutes at room temperature in the dark. The cells were then counterstained with 5 μg/ml propidium iodide (PI) (Sigma) in the presence of 100 μl of RNaseA (Sigma) for 30 minutes.

### Fluorescence measurements by flow cytometry

The FITC (green) and PI (red) fluorescence of individual cells in suspension induced by excitation with a 488-nm argon ion laser was measured using a FACScan flow cytometer (Becton-Dickinson, San Jose, CA, USA). The green and red fluorescence from each cell was separated and quantified using standard optics and Cell Quest software (Becton-Dickinson). Ten thousand cells were measured per sample. All experiments were repeated at least three times.

After γH2AX and DNA staining, the DNA content was represented on the x axis and the γH2AX content on the y axis using flow cytometry. The γH2AX in each cell cycle was determined, thereby allowing the relationships between cell kinetics and DNA damage induced by antitumor agents to be examined.

## Results

### Platinum Agents

#### CDDP

**Figure 1:**
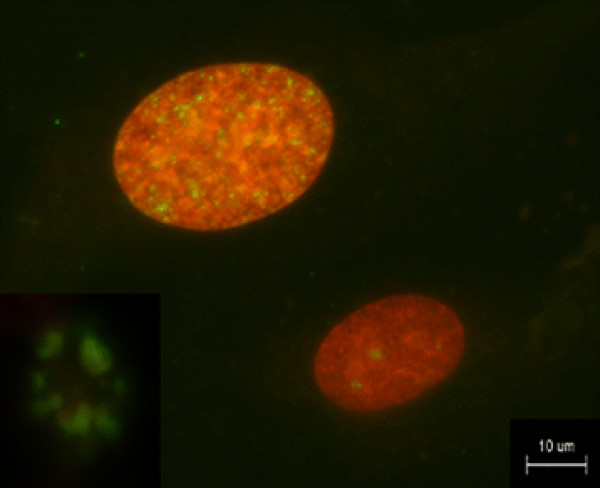
**Immunohistochemistry.** Representative microscopic images of OVISE cell line after exposure to 10 μg/ml CDDP for 24 h. γH2AX foci in a nuclear are stained green and red, respectively. An increase in green dots, indicating elevation of γH2AX, can be seen after exposure to CDDP. Thus, DNA damage is visually recognizable in each nucleus. Apoptotic bodies (insert) are distinguishable by the entire cell being intensely positive for γH2AX.

**Figure 2:**
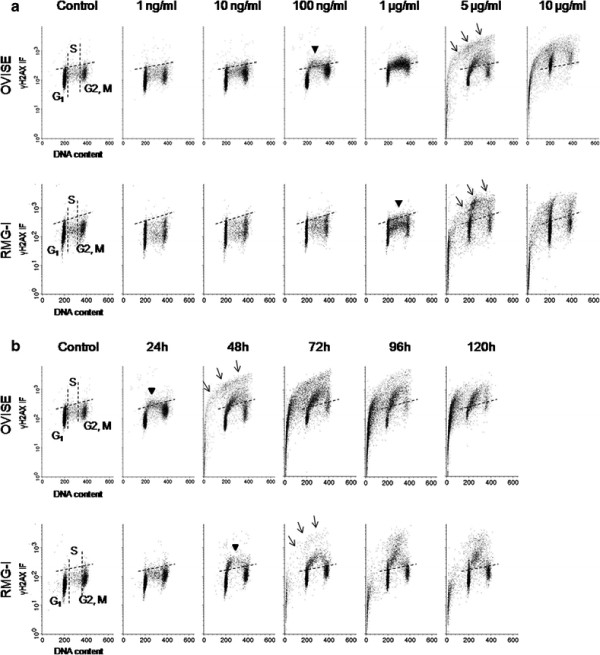
**Bivariate distributions (DNA content vs. γH2AX) of CCC cell lines, OVISE and RMG-I, treated with CDDP (Upper, OVISE; Lower, RMG-I).** The dotted lines indicate the upper level of γH2AX immunofluorescence for 95% of cells in the untreated (control) culture. Arrow heads indicate elevation of γH2AX that means DNA damage. Arrows indicate apoptotic cell populations with marked increase in γH2AX and gradual decrease in DNA. (A) Both cell lines treated with various concentrations of CDDP for 24 h. OVISE and RMG-1 cell lines exhibited DNA damage in S-phase cells at minimum concentrations of 100 ng/ml and 1 μg/ml, respectively. Both cell lines were subjected to DNA damage concentration-dependently at every cell cycle, and apoptosis was induced at concentrations of 5 μg/ml or higher. More cells in RMG-1 remained free of DNA damage as compared to OVISE. (B) Both cell lines were treated with 100 ng/ml, the minimum concentration inducing DNA damage in either cell line, for various reaction times. S-phase cells of OVISE showing DNA damage progressed to apoptosis after 48 h. In addition, S-phase arrest was observed. DNA damage was induced in S-phase cells of RMG-I after 48 h. Cells with DNA damage progressed to apoptosis after 72 h. Furthermore, cell-cycle arrest occurred in all cells.

OVISE cells showed an increase in the number of distinct green dots (γH2AX foci) after exposure to 10 μg/ml CDDP for 24 h, which indicates that CDDP caused DNA damage (Figure 1). Using folw cytometry, DNA damage was evident from the increase in γH2AX. After 24-hour treatment with CDDP, DNA damage in OVISE and RMG-I was seen gradually in the S phase at concentrations of 100 ng/ml and 1 ng/ml (Figure 2A). In both cell lines, treatment with 100 ng/ml or more CDDP for 24 hours caused DNA damage throughout the cell cycle. The cells with DNA damage gradually underwent apoptosis, as was evident by the presence of cells with markedly elevated γH2AX and fractional (sub-G1) DNA contents. In OVISE, DNA damage in the S and G2/M phases after treatment with 100 ng/ml CDDP was seen for 24 and 72 hours, respectively (Figure 2B). Although in RMG-I, 100 ng/ml CDDP caused DNA damage in the S phase, in other phases of the cell cycle it was not apparent even with longer treatment. In both cell lines, the cells with damaged DNA underwent apoptosis. The number of cells in the G2/M phase in OVISE decreased gradually indicating S-phase arrest. . On the other hand, in RMG-I showed G1 and G2/M-phases arrest. RMG-I was found to be less susceptible to DNA damage and subsequent apoptosis than OVISE.

#### CBDCA

**Figure 3:**
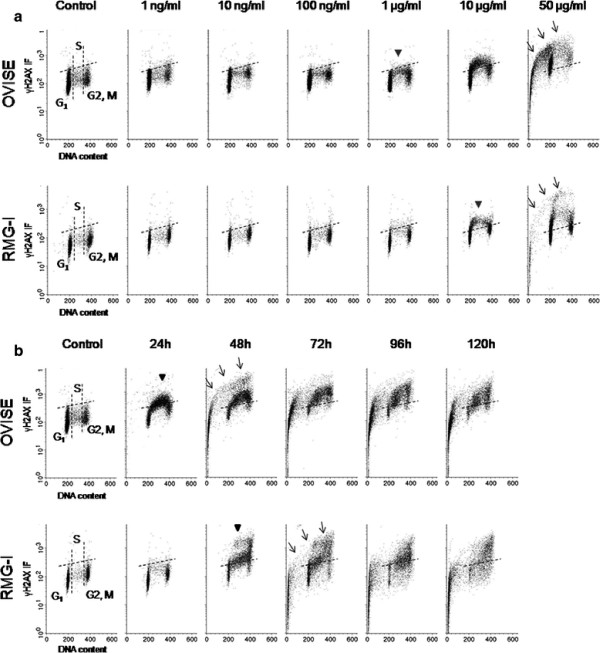
**Bivariate distributions (DNA content vs. γH2AX) of OVISE and RMG-I cell lines, treated with CBDCA (Upper, OVISE; Lower, RMG-I).** The dotted lines indicate control. Arrow heads and arrows indicate DNA damage and apoptosis, respectively. (A) Both cell lines treated with various concentrations of CBDCA for 24 h. DNA damage was observed in the S-phase cells at 1 μg/ml and 10 μg/ml concentrations in OVISE and RMG-1, respectively. DNA damage was found in both cell lines at every cell cycle as the concentration increased, and apoptosis occurred at a concentration of 50 μg/ml. More cells remained free of DNA damage in RMG-1 than in OVISE. (B) Both cell lines were treated with 1 μg/ml, the minimum concentration inducing DNA damage in either cell line, for various reaction times. In OVISE, S-phase cells with DNA damage progressed to apoptosis after 48 h. DNA damage was also found in G2/M-phase cells after 48 h, but most did not progress to apoptosis. S and G/2 M-phase arrests were observed. DNA damage was found in S and G2/M-phase cells after 48 h in RMG-I. The S-phase cells with DNA damage progressed to apoptosis 78 h later, but G2/M-phase cells showing DNA damage remained. S and G/2 M-phase arrests were observed.

DNA damage in the S phase was seen gradually after exposure to CBDCA for 24 hours in OVISE and RMG-I lines at 1 μg/ml and 10 μg/ml, respectively (Figure 3A). Subsequently, the cells with damaged DNA underwent apoptosis. Gradually both cell lines showed DNA damage in the G2/M phase and underwent apoptosis. OVISE showed S and G2/M- phases arrest, while RMG-I G2/M- phase arrest (Figure 3B).

#### PTX

**Figure 4:**
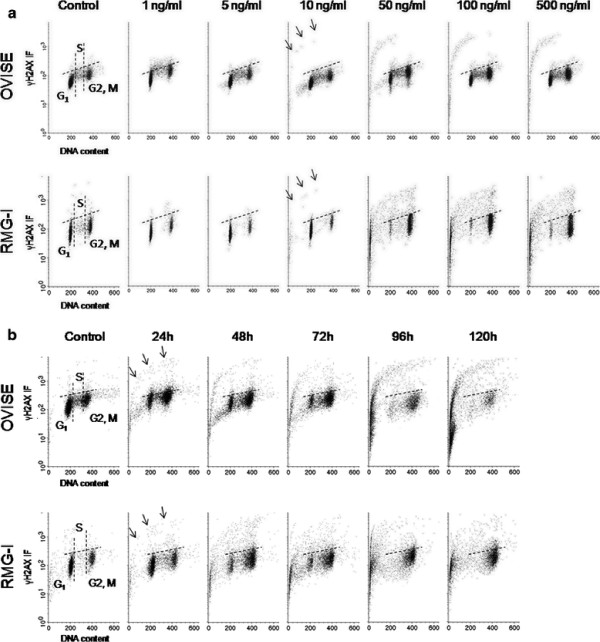
**Bivariate distributions (DNA content vs. γH2AX) of OVISE and RMG-I cell lines, treated with PTX (Upper, OVISE; Lower, RMG-I).** The dotted lines and arrows indicate control and apoptosis, respectively. (**A**) Both cell lines treated with various concentrations of PTX for 24 h. Both cell lines included cells that had progressed to apoptosis without direct DNA damage induced by a minimum concentration of 10 ng/ml. No concentration-dependent differences were observed at concentrations of 50 ng/ml or higher. (**B**) Both cell lines were treated with 10 ng/ml, the minimum concentration inducing DNA damage in either cell line, for various reaction times. G2/M-phase arrest occurred in both cell lines.

Exposure to 10 ng/ml or more PTX for 24 hours caused apoptosis without primary DNA damage in both cell lines. (Figure 4A). Although, further apoptotic effects were not seen at doses exceeding 50 ng/ml. PTX induced both cell lines G2/M-phase arrest, but some cells remained 120 hours after exposure without primary DNA damage (Figure 4B).

#### SN-38

**Figure 5:**
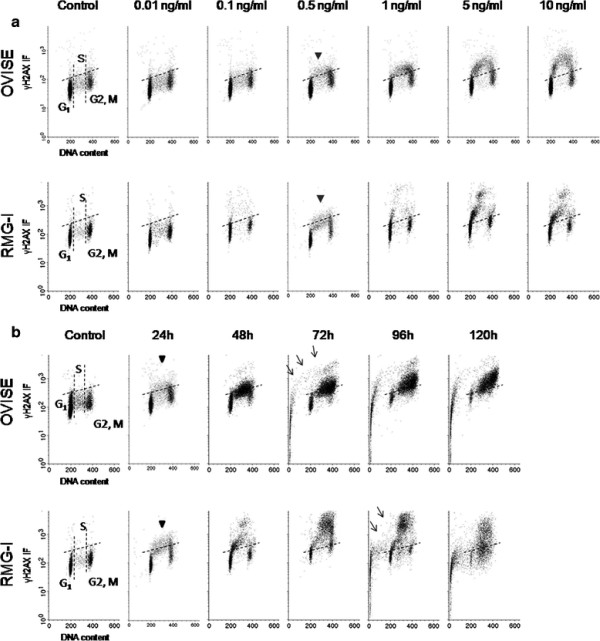
**Bivariate distributions (DNA content vs. γH2AX) of OVISE and RMG-I cell lines, treated with SN-38 (Upper, OVISE; Lower, RMG-I).** The dotted lines indicate control. Arrow heads and arrows indicate DNA damage and apoptosis, respectively. (**A**) Both cell lines treated with various concentrations of SN-38 for 24 h. Both cell lines exhibited DNA damage in S-phase cells at a minimum concentration of 0.5 ng/ml. (**B**) Both cell lines were treated with 0.5 ng/ml, the minimum concentration inducing DNA damage in either cell line, for various reaction times. S-phase cells with DNA damage started progressing to apoptosis after 72 and 96 h in OVISE and RMG-1, respectively. Both cell lines showed S-phase arrest, and large numbers of cells with DNA damage were still present even after 120 h.

After treatment with 0.5 ng/ml or more of SN-38 for 24 hours, both cell lines distinguished DNA damage in the S phase. Nevertheless even 10 ng/ml which is nearly the clinical maximum blood concentration did not cause apoptosis (Figure 5A). Apoptosis was seen barely 72 hours after exposure to 0.5 ng/ml SN-38 in a portion of OVISE while in RMG-I after 96 hours (Figure 5B). However, the cells with significant DNA damage remained in the S phase over 120 hours after exposure.

## Discussion

After CDDP administration, DNA damage was observed mainly in the S phase. It is reasonable to assume that the DNA was structurally altered by CDDP, leading to DNA replication fork arrest and ultimately resulting in apoptosis. This result was consistent with a known pharmacological effect of CDDP [12]. In RMG-I, apoptotic cells were minimally increased in the S phase, moreover the cells showing arrest in the G1 and G2/M phases without DNA damage were increased as compared with OVISE. Therefore, the results of the present study support the clinical experience that RMG-I is CDDP-resistant [11,13]. After CBDCA administration, DNA damage was seen in the S and G2/M phases in both cell lines. OVISE contained a remarkable cell population rescued from apoptosis and surviving with DNA damage. On the other hand, most RMG-I cells with DNA damage underwent apoptosis. These results suggest that cell lines respond differently to platinum agents, i.e., RMG-I was CDDP-resistant but responded to CBDCA. PTX directly induced apoptosis in M-phase cells but not via DNA damage, an observation consistent with a known pharmacological effect of PTX, i.e. microtubule inhibition [14]. PTX was confirmed to induce apoptosis through a p53-independent pathway; it was, therefore, expected to have an effect on CCC, in which the p53 mutation is rare [15,16]. The mechanism underlying the antitumor effect of PTX is G2/M arrest. Therefore, the combination with CBDCA, an agent inducing DNA damage, in G2/M-phase arrested cells might be effective, at least theoretically. As shown in this study, it is noteworthy that sensitivities to CDDP and CBDCA differed between the CCC cell lines. In practice, CCC is less sensitive to CBDCA/PTX treatment [4,6,17], which is the standard regimen for ovarian cancer. Since the effect of PTX was independent of both the concentration and the response time, these results raise the possibility that repeated administration of PTX at a low dose increases the antitumor effect more than a single administration. These findings support the results of the JGOG3016, i.e. that weekly CBDCA (AUC6)/PTX (80 mg/m^2^, weekly × 3) is more effective than tri-weekly CBDCA (AUC 6)/PTX (175 mg/m^2^) treatment [18].

On the other hand, after administration of SN-38, DNA damage occurred in S-phase cells, followed by apoptosis. This confirmed that SN-38 acts as a type I topoisomerase inhibitor [19]. Furthermore, it appears that SN-38 had an effect on the cell cycle because S-phase arrest continued for more than 120 hours. It is, therefore, possible that the improved administration method for SN-38 increases its antitumor effect. Cells rescued from apoptosis remained in S phase with DNA damage; consequently, the efficacy of combining SN-38 with CDDP, which induces DNA damage mainly in S-phase cells, was supported.

In conclusion, the present results suggest that an effective treatment for CCC with a slow growth rate and a low ratio of S-phase cells would be a combination of agents arresting the cell cycle, thereby causing accumulation of cells in the S phase or the G2/M phase, and agents specifically inducing DNA damage in S-phase cells. The method used in this study allows immunocytochemical detection of γH2AX, which indicates DNA damage even at very low concentrations and has high sensitivity in comparison with the comet assay. Employing this method, we were able to analyze relationships between anti-tumor effects and cell cycle perturbations. Therefore, γH2AX detection is a promising method of simply and rapidly identifying agents potentially effective against CCC.

## Misc

Eriko Takatori, Tadahiro Shoji, Seisuke Kumagai, Takashi Sawai, Akira Kurose, Toru Sugiyama contributed equally to this work.

## Competing interest

The authors have no conflicts of interest to report.

## Authors’ contributions

ET participated in all aspects of the study, from design to clinical and laboratory performance, and manuscript writing. TS participated in design, data analysis and drafting of the manuscript. AK participated in the design of the study and technical assistance. SK, TS and TS participated in design and analysis of clinical data. All authors have read and approved the manuscript.

## References

[B1] Sugiyama T Kamura T Kigawa J Terakawa N Kikuchi Y Kita T Suzuki M Sato I Taguchi K Clinical characteristics of clear cell carcinoma of the ovary Cancer 2000 88 2584 2589 10.1002/1097-0142(20000601)88:11<2584::AID-CNCR22>3.0.CO;2-5 10861437

[B2] McGuire WP Hoskins WJ Brady MF Kucera PR Partridge EE Look KY Clarke-Pearson DL Davidson M Cyclophosphamide and cisplatin compared with paclitaxel and cisplatin in patients with stage III and stage IV ovarian cancer N Engl J Med 1996 334 1 6 10.1056/NEJM199601043340101 7494563

[B3] Ozols RF Bundy BN Greer BE Fowler JM Clarke-Pearson D Burger RA Mannel RS DeGeest K Hartenbach EM Baergen R Gynecologic Oncology Group Phase III trial of carboplatin and paclitaxel compared with cisplatin and paclitaxel in patients with optimally resected stage III ovarian cancer: a Gynecologic Oncology Group study J Clin Oncol 2003 21 3194 3200 10.1200/JCO.2003.02.153 12860964

[B4] Sugiyama T Fujiwara K Govindan R Clear cell carcinoma of the ovary American Society of Clinical Oncology 2007 educational book 2007, Alexandria, VA 318 322

[B5] Takano M Kikuchi Y Yaegashi N Suzuki M Tsuda H Sagae S Udagawa Y Kuzuya K Kigawa J Takeuchi S Tsuda H Moriya T Sugiyama T Adjuvant chemotherapy with irinotecan hydrochloride and cisplatin for clear cell carcinoma of the ovary Oncol Rep 2006 16 1301 1306 17089053

[B6] Takano M Sugiyama T Yaegashi N Suzuki M Tsuda H Sagae S Udagawa Y Kuzuya K Kigawa J Takeuchi S Tsuda H Moriya T Kikuchi Y Progression-free survival and overall survival of patients with clear cell carcinoma of the ovary treated with paclitaxel-carboplatin or irinotecan-cisplatin: retrospective analysis Int J Clin Oncol 2007 12 256 260 10.1007/s10147-007-0670-1 17701003

[B7] Nishino K Aoki Y Amikura T Obata H Sekine M Yahata T Fujita K Tanaka K Irinotecan hydrochloride (CPT-11) and mitomycin C as the first line chemotherapy for ovarian clear cell adenocarcinoma Gynecol Oncol 2005 97 893 897 10.1016/j.ygyno.2005.03.009 15894369

[B8] Dickey JS Redon CE Nakamura AJ Baird BJ Sedelnikova OA Bonner WM H2AX: functional roles and potential applications Chromosoma 2009 118 683 692 10.1007/s00412-009-0234-4 19707781 PMC3094848

[B9] Fragkos M Jurvansuu J Beard P H2AX is required cell cycle arrest via the p53/p21 pathway Mol Cell Biol 2009 29 2828 2840 10.1128/MCB.01830-08 19273588 PMC2682023

[B10] Bonner WM Redon CE Dickey JS Nakamura AJ Sedelnikova OA Solier S Pommier Y γH2AX and cancer Nat Rev Cancer 2008 8 957 967 10.1038/nrc2523 19005492 PMC3094856

[B11] Itamochi H Kigawa J Sultana H Iba T Akeshima R Kamazawa S Kanamori Y Terakawa N Sensitivity to anticancer agents and resistance mechanisms in clear cell carcinoma of the ovary Jpn J Cancer Res 2002 93 723 728 10.1111/j.1349-7006.2002.tb01312.x 12079522 PMC5927055

[B12] Zwelling LA Kohn KW Mechanism of action of cis-Dichlorodiammineplatinum (II) Cancer Treat Rep 1979 63 1439 1444 387221

[B13] Okuma Y Kiguchi K Koshitaka Y Okamura A Ishiwata I Kondo H Ishizuka B Tadokoro M Correlation between expression of oncogene products and resistance to anticancer drugs in cultured ovarian cancer cell lines Hum Cell 2003 16 131 139 10.1111/j.1749-0774.2003.tb00145.x 15005244

[B14] Rowinsky EK Donehower RC Jones RJ Tucker RW Microtubule changes and cytotoxicity in leukemic cell lines treated with Taxol Cancer Res 1988 48 4093 4100 2898289

[B15] Takahashi M Kigawa J Minagawa Y Itamochi H Shimada M Kamazawa S Sato S Akeshima R Terakawa N Sensitivity to paclitaxel is not related to p53-dependent apoptosis in ovarian cancer cells Eur J Cancer 2000 36 1863 1868 10.1016/S0959-8049(00)00183-0 10974635

[B16] Ho ES Lai CR Hsieh YT Chen JT Lin AJ Hung MH Liu FS p53 mutation is infreq1uent in clear cell carcinoma of the ovary Gynecol Oncol 2001 80 189 193 10.1006/gyno.2000.6025 11161858

[B17] Enomoto T Kuragaki C Yamasaki M Sugita N Otsuki Y Ikegami H Matsuzaki M Yamada T Wakimoto A Murata Y Is clear cell carcinoma and mucinous carcinoma of the ovary sensitive to combination chemotherapy with paclitaxel and carboplatin? Proc Am Soc Clin Oncol 2003 22 447(#1797)

[B18] Katsumata N Yasuda M Takahashi F Isonishi S Jobo T Aoki D Tsuda H Sugiyama T Kodama S Kimura E Ochiai K K Noda Japanese Gynecologic Oncology Group Dose-dense paclitaxel once a week in combination with carboplatin every 3 weeks for advanced ovarian cancer : a phase 3, open-label, randomized controlled trial Lancet 2009 374 1331 1338 10.1016/S0140-6736(09)61157-0 19767092

[B19] Hsiang YH Liu LF Wall ME Wani MC Nicholas AW Manikumar G Kirschenbaum S Silber R Potmesil M DNA topoisomerase-I mediate DNA cleavage and cytotoxicity of camptothecin analogs Cancer Res 1989 49 4385 4389 2545341

